# Current evidence and strategies for bridging therapy in CD19-directed chimeric antigen receptor T-cell therapy for relapsed/refractory large B-cell lymphomas

**DOI:** 10.1007/s00277-026-06950-0

**Published:** 2026-03-23

**Authors:** Jie Lv, Yan Xie, Chen Zhang, Jili Deng, Yuqin Song, Jun Zhu

**Affiliations:** 1 Key Laboratory of Carcinogenesis and Translational Research (Ministry of Education/Beijing), Department of Lymphoma, Peking University Cancer Hospital & Institute, Beijing, 100142 China; 2 Department of Medical Oncology, Sichuan Clinical Research Center for Cancer, Sichuan Cancer Center, School of Medicine, Sichuan Cancer Hospital and Institute, University of Electronic Science and Technology of China, Chengdu, 610041 Sichuan China

**Keywords:** Chimeric antigen receptor, Bridging therapy, Relapsed or refractory, Large B-cell lymphoma

## Abstract

CD19-directed chimeric antigen receptor T-cell (CAR T-cell) therapy has markedly improved the prognosis of patients with relapsed or refractory large B-cell lymphoma (R/R LBCL). However, disease progression during the manufacturing period remains a major barrier to successful treatment. Bridging therapy (BT), defined as anti-lymphoma treatment administered between leukapheresis and lymphodepleting chemotherapy, serves two primary purposes: to prevent disease progression ensuring eligibility for CAR T-cell infusion, and to modulate the immune microenvironment to potentially enhance CAR T-cell efficacy or mitigate its toxicity. This review provides a comprehensive overview of current strategies and clinical evidence regarding BT in the context of CAR T-cell therapy. We systematically examine the efficacy and safety profiles of various BT strategies, including chemotherapy, targeted or immunotherapy agents, and radiotherapy. Furthermore, we summarize and compare findings from pivotal clinical trials and real-world studies, offering insights into the practical application and outcomes of BT in diverse clinical settings. Unresolved questions remain, including the optimal implementation of bispecific antibodies as BT regimens, the timing and duration of Bruton’s tyrosine kinase inhibitors administration, the safety and efficacy of reusing polatuzumab vedotin in previously exposed patients, and the standardization of radiotherapy protocols. In conclusion, the rational selection and application of BT strategies hold promise for improving the clinical outcomes of R/R LBCL patients undergoing CAR T-cell therapy.

## Introduction

Large B-cell lymphoma (LBCL) is the most common subtype of non-Hodgkin lymphoma, characterized by biologically aggressive behavior. Approximately one-third of patients experience primary refractory disease or relapse after achieving complete response (CR) [1]. In recent years, CD19-directed chimeric antigen receptor (CAR) T-cell therapy have emerged as a standard treatment option in the second-line or later settings for patients with relapsed or refractory (R/R) LBCL, demonstrating substantial clinical efficacy with high rates of objective and complete responses.

However, early clinical trial data have shown that approximately 7% of patients experienced disease progression or death during the interval required for CAR T-cell manufacturing, thereby losing the opportunity to receive treatment [2]. Furthermore, a high tumor burden prior to CAR T-cell infusion has been associated with lower complete response rate (CRR) and shorter overall survival (OS) following therapy [3, 4]. These findings underscore the need for appropriate anti-lymphoma treatment during the manufacturing period—commonly referred to as bridging therapy (BT)—which is considered a critical measure to ensure therapeutic success.

With the growing body of research on BT prior to CAR T-cell therapy in patients with LBCL, the range of available bridging strategies has expanded significantly. Accordingly, there is an urgent need to systematically review current approaches and clinical practices. This review aims to clarify the fundamental concepts of BT, provide an overview of the diverse strategies employed, and analyze their implementation in pivotal clinical trials and real-world studies. By identifying key areas for future investigation, this work seeks to optimize the selection of bridging strategies and ultimately contribute to improving outcomes for patients with LBCL.

## Definition and purpose of BT

BT is defined as anti-lymphoma treatment administered between leukapheresis and lymphodepleting chemotherapy during CAR T-cell manufacturing. It should be distinguished from holding therapy, which is given before leukapheresis—specifically, during the period between production slot assignment and leukapheresis. The duration of BT is determined by the CAR T-cell production timeline and this interval may vary slightly among different CAR T-cell constructs [5].

The primary objective of BT is to maintain disease stability and reduce tumor burden, thereby preventing rapid disease progression that could preclude CAR T-cell infusion. Additionally, BT may also modulate the immune microenvironment, potentially mitigating CAR T-cell related toxicities and enhancing the efficacy of subsequent CAR T-cell therapy.

Evidence indicates that high tumor burden can negatively influence the efficacy of CAR T-cell therapy through multiple mechanisms [6]. Elevated tumor burden promotes the release of IL-1, IL-6, and granulocyte colony-stimulating factor, which suppress systemic T-cell immune responses, while also fostering a more immunosuppressive tumor microenvironment (TME)—characterized by increased myeloid-derived suppressor cells (MDSCs) and regulatory T cells (Tregs)—thereby impairing CAR T-cell function [6]. Reducing tumor burden may therefore enhance CAR-T efficacy and improve patient outcomes. Lyu et al. reported that effective debulking chemotherapy improved both short-term objective response rate (ORR) and long-term OS after CAR T-cell therapy in high–tumor-burden patients, resulting in outcomes comparable to those of patients with low tumor burden [7]. Analysis of the prospective safety expansion cohort 5 of the ZUMA-1 study showed that patients with lower baseline tumor burden achieved more durable responses, with a median duration of response of 25.8 months versus 11.1 months in those with higher tumor burden [8].

The TME is also a critical determinant of the efficacy of CD19 CAR T-cell therapy. Locke et al. reported that low pre-treatment CD19 expression and a high stromal and immunosuppressive index (SII) gene expression signature were associated with inferior event-free survival (EFS) after axi-cel, whereas a B-cell gene expression signature correlated with improved EFS [9]. Similarly, Hirayama et al. demonstrated that the pre-treatment TME influences CAR-T effectiveness across multiple dimensions, including transcriptomic profiles, immune-cell density, histologic features, and spatial organization. For emalple, higher CD4^+^ T-cell density was observed in CR patients, along with a lower proportion of interspersed immune-infiltrated regions and a higher proportion of hypocellular/fibrotic areas [10].

Moreover, patients who achieve CR/PR after BT generally experience higher remission rates after CAR T-cell infusion, along with longer progression-free survival (PFS) and OS. In the study by Roddie et al., responders to BT (CR/PR) had a 42% lower risk of progression or death than non-responders (SD/PD), with significantly higher 1-year PFS rates (50.1% vs. 29.7%, *P* = 0.001) and OS rates (63.2% vs. 45.9%, *P* = 0.001) [11]. Similarly, an Australian study of tisagenlecleucel showed that non-responders had nearly twice the risk of disease progression compared with responders [12]. These findings suggest that the “BT response” may serve as a prognostic indicator of subsequent CAR-T efficacy and long-term survival.

## BT strategies

BT strategies can broadly be classified into three categories: (1) chemotherapy; (2) targeted or immunotherapy agents; and (3) radiotherapy [13]. In Table 1, we attempted to provide a summary of the different BT strategies. The selection of a BT strategy exhibits significant heterogeneity due to tumor biological characteristics and inter-patient variability [14]. Key considerations include disease subtype, prior treatment responses and residual toxicities, the anticipated efficacy and safety of the proposed BT and its potential impact on the TME, as well as the patient’s current tumor burden and involvement of critical organs. An ideal BT strategy should satisfy the following criteria: first, maintain disease control to preserve eligibility for CAR T-cell therapy; and second, avoid severe complications (e.g., infection, bleeding, or organ decompensation) that could delay CAR T-cell infusion.

**Table 1:** Summary of BT strategies

*BT strategies*	Specific bridging regimens	Additional notes
*Chemotherapy*	Platinum-based regimens	Limited efficacy; Hematologic/organ toxicities
	Bendamustine	Lymphotoxic effects; Pre- vs. post-leukapheresis use and washout requirements; Impact on TME
	BBB-penetrating agents (e.g., HD-MTX)	No consensus; Further research needed
*Targeted/immunotherapy agents*	Rituximab (anti-CD20 mAb)	Resistance after prior rituximab; Rituximab-related AEs
	BsAbs (CD20×CD3)	Shared T-cell resources with CAR T-cells; Risk of immune exhaustion; Effective but lacking long-term survival data; Potential toxicities
	BTKi	Promising BT option; Timing and duration of BTKi use require further study
	Pola (anti-CD79b ADC)	Good disease control pre-CAR-T and favorable post-CAR-T survival; Concerns about the efficacy of re-treatment
	TAFA (anti-CD19 mAb)	Post-TAFA progression should not preclude CD19 CAR T-cell therapy; Further research needed
	Lonca (anti-CD19 ADC)	May not adversely affect subsequent CD19 CAR T-cell efficacy or safety; Further research needed
	LEN	Enhanced CAR T-cell activity in vitro/in vivo models
	Other agents (e.g., EZH2 inhibitors, BCL-2 inhibition agents, corticosteroid-only regimen)	Further research needed
*Radiotherapy*	-	High efficacy and favorable safety; No consensus on optimal radiation field design, dose, or fractionation

Abbreviations: *AEs* adverse events, *ADC* antibody-drug conjugate, *BBB* blood–brain barrier, *BsAbs* bispecific antibodies, *BT* bridging therapy, *BTKi* Bruton’s tyrosine kinase inhibitor, *HD-MTX* high-dose methotrexate, *LEN* lenalidomide, *Lonca* loncastuximab tesirine, *mAb* monoclonal antibody, *Pola* polatuzumab vedotin, *TAFA* tafasitamab, *TME* tumor microenvironment

### Bridging chemotherapy

Chemotherapy, with or without the addition of targeted agents, remains the most widely utilized bridging regimen [13, 15, 16]. Data from the U.S. Lymphoma CAR-T Consortium indicate that 54% of patients requiring BT received chemotherapy [15]. Among the available options, classical platinum-based regimens—such as R-DHAP (rituximab, dexamethasone, high-dose cytarabine, cisplatin), R-ICE (rituximab, ifosfamide, carboplatin, etoposide), and R-GDP (rituximab, gemcitabine, dexamethasone, cisplatin)—are commonly employed as bridging chemotherapy. These regimens are often selected for patients who have failed first-line therapies such as R-CHOP (rituximab, cyclophosphamide, doxorubicin, vincristine, prednisone), among whom the ORR remain suboptimal, typically ranging from 30% to 50%. In addition to their limited efficacy in this population, platinum-based regimens are associated with considerable hematologic and organ toxicities, necessitating close clinical monitoring [17, 18].

As a chemotherapeutic agent, bendamustine has also been investigated as a bridging regimen. Importantly, the timing of its use appears to have a significant impact on clinical outcomes. Because bendamustine exerts prolonged lymphotoxic effects, administering it prior to leukapheresis—particularly as holding therapy—may be associated with inferior outcomes after CAR T-cell therapy. In contrast, its use as BT after leukapheresis appears to be safe and feasible. In a multicenter European study involving seven centers, the bendamustine-exposed group prior to leukapheresis demonstrated significantly poorer outcomes compared with the bendamustine-naïve group, including lower ORR (53% vs. 72%; *P* < 0.01), shorter PFS (3.1 months vs. 6.2 months; *P* = 0.04), and reduced OS (10.3 months vs. 23.5 months; *P* = 0.01) [19]. Further analyses indicated that a washout period of < 9 months before leukapheresis was associated with even lower ORR, PFS, and OS [19]. However, among patients who had already undergone leukapheresis, a study by Iacoboni et al. showed no significant difference in CRR between those who did and did not receive bendamustine-containing BT (*P* > 0.05). Moreover, the incidence of ≥ grade 3 cytokine release syndrome (CRS) and ≥ grade 3 immune effector cell-associated neurotoxicity syndrome (ICANS) was not increased (*P* > 0.05). Peak CAR T-cell expansion and the median time to peak were similar between the benda and non‐benda BT groups (*P* > 0.05) [20]. In addition to its lymphotoxic effects, bendamustine also influences the TME. Prolonged treatment with bendamustine–rituximab has been shown to induce pyroptotic cell death in diffuse large B-cell lymphoma (DLBCL) through activation of the cGAS–STING signaling axis. Engagement of this pathway promotes the secretion of inflammatory mediators and enhances the expression of MHC molecules, collectively fostering a more immunologically active, “hot” TME. Consequently, the bendamustine–rituximab regimen can stimulate endogenous immunity and contribute to antitumor activity [21].

Patients with primary or secondary Central Nervous System (CNS) involvement generally have poor prognoses, whereas CAR T-cell therapy offers the potential for improved outcomes in this high-risk population [22, 23]. When selecting BT for patients with CNS disease, the ability of agents to penetrate the blood–brain barrier is a key consideration. As no dedicated studies have evaluated BT specifically for CNS involvement, current practice is largely informed by systemic treatment strategies for CNS lymphomas. High-dose methotrexate (HD-MTX)-based regimens are supported by most available evidence [24, 25]. In the cohort reported by Shumilov et al., 80% (12/15) of patients with CNS disease received BT, and 42% (5/12) were treated with MTX-containing systemic regimens [22].

### Bridging with targeted or immunotherapy agents

#### Rituximab

Rituximab, a CD20-targeting monoclonal antibody, can be administered as monotherapy or in combination with chemotherapy or other targeted agents. In the JULIET trial, 54% of bridging regimens contained rituximab [16], while in the TRANSFORM trial, 63% of patients received rituximab-based chemotherapy regimens, including R-DHAP, R-ICE, and R-GDP [26]. Similarly, a retrospective analysis of axi-cel and tisa-cel recipients reported that 69% of bridging regimens incorporated rituximab [27].

However, when selecting rituximab for bridging purposes, careful consideration is required. Rituximab resistance following prior exposure may develop through several biological mechanisms. A key factor is the reduction or loss of CD20 expression on lymphoma cells—arising from transcriptional downregulation, antigen internalization, or clonal selection—which ultimately compromises antibody–target binding [28, 29]. In addition, rituximab-associated adverse events, such as infusion-related reactions [30], hepatitis B virus reactivation [31], and an increased risk of infections [32], further constrain its use in BT.

#### Bispecific antibody

Bispecific antibodies (BsAbs) used in the treatment of LBCL predominantly target CD20 and CD3 (e.g., mosunetuzumab, epcoritamab, and glofitamab). When BsAbs are considered for use as a bridging regimen, some issues require careful evaluation. A key concern is that BsAb and CAR T-cells rely on shared T-cell–dependent resource. BsAb recruit and activate endogenous polyclonal T cells through CD3 engagement [33], which may decrease the quantity and functional fitness of T cells available for leukapheresis and subsequently impair CAR T-cell manufacturing. In addition, several studies have raised concerns that BsAb may induce immune exhaustion, thereby diminishing CAR T-cell proliferative capacity and antitumor activity [34]. In an in vitro study, Philipp et al. demonstrated that continuous exposure to a BsAb for 28 days led to a progressive loss of T-cell function over time [35].

However, preliminary clinical evidence suggests that prior exposure to BsAb—including their use as BT—does not seem to substantially impair the efficacy of subsequent CAR T-cell therapy. A retrospective analysis from the DESCAR-T registry showed that patients who experienced disease progression following prior BsAb therapy still achieved an ORR of 92.9% and a CRR of 50.0% with subsequent CAR T-cell therapy [36]. A recent multicenter study from Germany included 40 patients across six centers, among whom 20 received glofitamab as BT. At the first response assessment following CAR T-cell infusion, 13 patients (59.1%) achieved CR and 6 (27.3%) achieved partial remission (PR), resulting in an ORR of 86.4% [37]. Nevertheless, these findings require confirmation through longer-term survival follow-up. In addition, when selecting BsAb as BT, their potential toxicities—such as CRS, hematologic toxicity, and infections [38]—must be taken into consideration, as these may delay timely CAR-T infusion.

Given the effects of BsAb on CAR T-cell therapy, the interval between BsAb exposure and either T-cell leukapheresis or CAR T-cell infusion may merit consideration. Philipp et al. reported that introducing treatment-free intervals after BsAb therapy can “rejuvenate” T cells, restoring their functional and transcriptional profiles [35]. Crochet et al. evaluated a 50-day cutoff before leukapheresis, comparing shorter versus longer wash-out intervals. They found no significant differences in CAR T-cell therapy efficacy (ORR, CRR) or survival outcomes (PFS, OS) between the two groups, a finding potentially attributable to the relatively short half-life of BsAb (10–20 days) [39]. Nevertheless, more studies are needed to better define the optimal wash-out duration following BsAb therapy.

#### Bruton’s tyrosine kinase inhibitor

Bruton’s tyrosine kinase inhibitor (BTKi) represents a promising BT option, with both preclinical and clinical evidence supporting their synergistic effect in combination with CAR T-cell therapy [5]. BTKi have been shown to enhance CAR T-cell expansion and antitumor efficacy while also mitigating treatment-related toxicities. Among the current clinical evidence on BTKi combined with CAR T-cell therapy, data on ibrutinib and zanubrutinib are relatively more robust, whereas studies involving orelabrutinib and acalabrutinib remain limited.

Fraietta et al. demonstrated that ibrutinib enhances CAR T-cell expansion and reduces the expression of programmed cell death 1 on T cells, both of which were positively correlated with clinical response [40]. Gauthier et al. demonstrated that in patients with chronic lymphocytic leukemia (CLL) who had progressed on or failed to respond to prior ibrutinib therapy, the combination of CAR T-cells with ibrutinib could still provide substantial clinical benefit. Compared with patients who did not receive concurrent ibrutinib, this combination approach was associated with reduced severity of CRS and higher rates of minimal residual disease (MRD) negativity based on IGH sequencing [41]. The TARMAC phase II trial (*n* = 20, mantle cell lymphoma (MCL) patients) further demonstrated that, regardless of prior BTKi exposure or the presence of high-risk clinical or molecular features, the combination of BTKi and CAR T-cell therapy remained effective. At 4 months post-infusion, both the ORR and CRR reached 80%. Longer pre-infusion ibrutinib exposure was associated with more robust expansion, reflected by higher peak levels and an increased area under the curve (AUC) [42].

In addition, recent study suggested that the combination of zanubrutinib and tislelizumab may enhance the efficacy of CAR T-cell therapy, with 2-year PFS and OS rates of 68% and 76%, respectively [43]. Similarly, Wang et al. reported that the combination of CAR T-cell therapy and zanubrutinib resulted in CR in all patients, with a 3-month ORR of 100% following CAR T-cell infusion [44]. In another retrospective study by Xu et al., 17 patients received zanubrutinib in combination with CAR T-cells, achieving an ORR of 88.2%, including a CRR of 70.5% [45].

In an open-label phase I/II clinical trial (NCT04257578), acalabrutinib was administered continuously during the bridging phase, cell therapy phase, and following axi-cel infusion. On day 30 post-infusion, the ORR and CRR were 93% and 71%, respectively [46]. Luo et al. reported from both in vivo and in vitro experiments that ibrutinib, zanubrutinib, and acalabrutinib help alleviate CAR T-cell exhaustion by inhibiting CD3-ζ phosphorylation in CAR constructs and T-cell receptor, as well as by downregulating genes involved in T-cell activation signaling pathways. Moreover, BTKi lowered the production of IL-6 and tumor necrosis factor-α, indicating their potential utility in mitigating CRS [47].

The optimal timing for integrating BTKi with CAR T-cell therapy has garnered increasing attention, with key considerations including when to initiate BTKi, whether immediate administration after CAR T-cell infusion is necessary, and how long maintenance therapy should be continued. Current exploratory evidence is derived primarily from studies in CLL and MCL, and no unified consensus has yet been established. In the study by Fraietta et al., patients with CLL received at least five cycles of ibrutinib before CAR T-cell infusion [40]. In the trial reported by Gauthier et al., CLL patients initiated daily ibrutinib treatment at least two weeks prior to leukapheresis and continued therapy until at least three months after CD19 CAR T-cell infusion; dose reductions were permitted in cases of toxicity [41]. In the TARMAC trial, patients with MCL received ibrutinib for at least seven days before leukapheresis. Ibrutinib was discontinued in patients who achieved CR at six months post-infusion and had peripheral blood MRD levels < 10^− 5^ by flow cytometry [42]. In the study by Shen et al., patients with LBCL received daily zanubrutinib beginning at the time of leukapheresis and continued through day 28 following CAR T-cell infusion. Patients who achieved remission remained on zanubrutinib for an additional three months [43].

#### Polatuzumab vedotin

Polatuzumab vedotin (Pola) is a CD79b-targeting antibody–drug conjugate, and incorporating Pola into bridging regimen may represent a reasonable alternative to conventional platinum‐based protocols [48–50]. Emerging data demonstrate that Pola‐containing bridging regimen achieves satisfactory disease control before CAR T-cell infusion and is associated with favorable post–CAR T-cell therapy survival outcomes.

In a multicenter retrospective analysis from Germany involving 26 institutions, 41 patients (51.2%) who received Pola-based BT had a 1-year OS rate of 58.5% from the initiation of treatment [49]. The German Lymphoma Alliance/German Registry for Stem Cell Transplantation (GLA/DRST) study (*n* = 356) confirmed that the ORR among patients receiving Pola-based bridging (34%) was significantly higher than the overall bridging response rate (22%) [51]. A real-world study from the United Kingdom (UK) similarly demonstrated that the rituximab-bendamustine-polatuzumab regimen, when used as BT, resulted in a notable ORR of 42% [11]. Notably, a chemotherapy-free combination of Pola plus rituximab yielded a 40% ORR in patients deemed unfit for cytotoxic chemotherapy [49].

Roddie et al. proposed that the notable efficacy of Pola may be partly attributed to the absence of prior exposure to CD79b-targeted therapies, thereby reducing the likelihood of preexisting resistance [11]. However, with the increasing clinical evidence supporting the use of polatuzumab in the first line [52], questions arise as to the efficacy of re-treatment as BT. Mechanistic studies investigating resistance to Pola have identified several contributing factors, including decreased CD79b expression, increased expression of multidrug resistance proteins, and alterations in apoptotic regulatory pathways [53, 54]. These findings highlight the potential challenges of reusing Pola in patients previously exposed to the agent. While clinical data on the safety and efficacy of Pola re-challenge—particularly as BT after prior exposure—remain limited, preliminary exploratory studies suggest that Pola may retain antitumor activity even in the presence of acquired resistance, as demonstrated in preclinical DLBCL models [55].

#### CD19-targeted therapies

The suitability of anti-CD19 therapies—such as tafasitamab (TAFA) and loncastuximab (Lonca)—as BT prior to CD19 CAR T-cell infusion remains uncertain, and some questions have yet to be clarified. First, it remains unclear whether prior exposure to anti-CD19 agents affects subsequent CD19 antigen expression. A small retrospective exploratory analysis from the phase II L-MIND study, using immunohistochemistry, whole-exome sequencing, and RNA sequencing, demonstrated that CD19 expression levels remained stable and no genomic alterations were detected before and after TAFA treatment [56]. Second, the relationship between baseline CD19 expression density and the efficacy of subsequent CD19 CAR T-cell therapy is also under investigation. Some studies suggest that low CD19 surface density prior to CAR T-cell infusion correlates with disease progression following treatment [57]. In contrast, findings from the JULIET and ZUMA-2 trials reported no significant difference in ORR between patients with confirmed CD19 expression and those with low or undetectable expression [16, 58]. Third, while evaluation of CD19 expression prior to CAR T-cell therapy—particularly in patients previously treated with anti-CD19 agents—is generally recommended, no standardized methods or thresholds for CD19 detection have been established to date [59].

##### Tafasitamab

TAFA is a humanized monoclonal antibody targeting CD19 [60–62]. Currently, there is no definitive clinical evidence supporting the use of TAFA as a bridging agent. In a preclinical study, Sakemura et al. demonstrated that in vitro exposure of CD19^+^ cell lines to TAFA, followed by removal of unbound antibody, did not impair CAR T-cell cytotoxicity. In murine models, TAFA preconditioning was associated with a lower incidence and severity of CRS, and led to enhanced antitumor efficacy and prolonged OS compared with CAR T-cell therapy alone [63]. Additionally, some clinical observations suggest that prior exposure to TAFA may not preclude the efficacy of subsequent CD19-targeted CAR T-cell therapy and could still confer survival benefit [64, 65]. Whether TAFA can be safely and effectively incorporated into bridging regimens remains to be determined through further prospective studies.

##### **Loncastuximab tesirine**

Loncastuximab tesirine (Lonca) is a CD19-targeted antibody–drug conjugate [66] that has been approved by the U.S. FDA for the treatment of R/R LBCL after at least two prior lines of systemic therapy. Emerging evidence suggests that prior exposure to Lonca may not adversely affect the efficacy of subsequent CD19 CAR T-cell therapy. In a study involving 14 patients previously treated with Lonca, the ORR after CAR T-cell therapy was 50% [67]. In another analysis from the Center for International Blood and Marrow Transplant Research (CIBMTR) registry, 16 patients received Lonca as BT or as the last line of treatment before CAR T-cell infusion. Among these patients, the post-CAR-T CRR was 44% and the PR rate was 19%; the 1-year OS and PFS rates were 33% and 28%, respectively [68]. A proposed explanation for the preserved activity of CD19 CAR T-cells after Lonca exposure is the difference in their epitope specificity: whereas most current CD19 CAR T-cell constructs recognize the FMC63 epitope, Lonca targets the RB4 epitope [69]. Regarding safety, a study by Thapa et al. reported that prior exposure to Lonca did not alter the expected safety profile of subsequent CAR T-cell therapy, with toxicities mainly limited to grade 1–2 CRS and ICANS—consistent with previously reported CD19 CAR T-cell data [67]. To further evaluate the safety and efficacy of Lonca in combination with rituximab as a bridging regimen, a phase 2 study (NCT06788964) has been initiated, and its results are anticipated. However, questions regarding the optimal timing of Lonca administration and the potential need for a wash-out period before CAR T-cell infusion remain unanswered and require additional study.

#### Lenalidomide

Lenalidomide (LEN), an immunomodulatory agent, has been shown in both in vitro and in vivo preclinical models to enhance CAR T-cell function, providing a rationale for its use as bridging regimen [70]. On the one hand, LEN polarizes CD8^+^ CAR T-cells toward an early differentiation stage and a Th1 phenotype, significantly reducing CAR T-cell exhaustion and promoting cell expansion. On the other hand, LEN remodels the TME to augment CAR T-cell infiltration into tumor sites. This synergistic effect was confirmed in a DLBCL mouse model, where the combination of LEN and CAR T-cell resulted in a marked reduction in tumor burden and prolonged survival compared with CAR T-cell monotherapy [70].

#### Other agents

Studies investigating the synergistic mechanisms of other small-molecule targeted agents in conjunction with CAR T-cell therapy have provided novel insights for BT. EZH2 inhibitors have been shown to re-activate immune synapse functions between B‐cell lymphoma cells and T lymphocytes, thereby enhancing tumor sensitivity to T‐cell–based immunotherapy. EZH2 inhibition enhances killing activity and prevents T cell exhaustion [71]. Moreover, BCL‐2 inhibition has been demonstrated to augment the cytolytic activity of CAR T-cells [72]. These mechanisms warrant further elucidation and be validated in clinical cohorts.

The advantages of corticosteroids as BT include their ability to rapidly reduce tumor burden, modulate immune, and have relatively low toxicity. In lymphoma, corticosteroids exert direct effects through the glucocorticoid receptor on key targets within the B-cell receptor (BCR) signaling pathway, thereby suppressing oncogenic BCR signal transduction [73]. Corticosteroid-only regimen may therefore be considered as one of the potential bridging approaches. In the study by Pinnix et al., 9% (4/45) of patients receiving BT were treated with high-dose corticosteroids alone [74]. In the pivotal ZUMA-7 trial, where corticosteroids were the only permitted BT, 36% (65/180) of patients received steroid bridging [75].

### Bridging radiotherapy

Radiotherapy differs from systemic chemotherapy or targeted/immunotherapy by delivering ionizing radiation to selectively damage cancer cells. As a local therapy, it achieves high response rates in patients refractory to chemotherapy and without prior radiation [76–78].

#### Effectiveness

Retrospective studies indicate that bridging radiotherapy (BRT) may provide superior survival outcomes compared with systemic BT. A multicenter study from 12 UK institutions reported that BRT can be safely and effectively administered even in patients with advanced, high-risk disease (elevated LDH, IPI ≥ 3), with low CAR T-cell therapy dropout rates and favorable survival outcomes. The 1-year PFS rates for the BRT group, the combined-modality bridging group, and the systemic BT group were 56%, 47%, and 43%, respectively; corresponding 1-year OS rates were 62%, 51%, and 54% [79]. Another study demonstrated that BRT with or without chemotherapy was associated with improved outcomes compared with chemotherapy-only bridging. The 30-day ORR was 82.8% vs. 45.2% (*P* = 0.0025), and the 1-year PFS was 46.9% vs. 22.6% (*P* = 0.0356). The benefit was particularly notable in patients with bulky disease, where 6-month PFS was 50.8% vs. 16.7% (*P* = 0.0369), and 1-year OS was 56.3% vs. 33.3% (*P* = 0.0236) [80]. At MD Anderson, BRT achieved higher ORR (100% vs. 67%) and CRR (82% vs. 38%), as well as superior 1-year PFS (44% vs. 25%), compared with systemic BT [74].

The mechanisms through which BRT enhances CAR-T efficacy are likely multifactorial. First, radiotherapy can directly control disease through local tumor debulking, thereby improving clinical outcomes. In addition, its abscopal effects and its capacity to modulate the TME also contribute beneficially to treatment response. In a murine model, Kostopoulos et al. demonstrated that combining radiotherapy with CAR T-cell therapy not only produced additive growth inhibition at irradiated tumor sites but also significantly enhanced antitumor activity at non-irradiated lesions. Notably, a low-dose fractionated regimen (4 Gy × 2 fractions) was particularly effective in inducing abscopal effects [81]. Chen et al. proposed a three-stage mechanistic framework—“initiation, amplification, and reinforcement”—to describe how radiotherapy facilitates durable systemic tumor control [82]. At the level of the TME, radiotherapy can promote immunogenic cell death [83], activate the cGAS–STING pathway [83], and upregulate chemokines and adhesion molecules [84], thereby converting an immunosuppressive TME into an immune-permissive one. This transition enhances CAR T-cell homing, infiltration, and effector function. However, radiotherapy may also upregulate PD-L1 expression and increase the infiltration of Tregs and MDSCs [85], indicating that its impact on TME includes both immune-stimulatory and immune-suppressive components.

#### Safety

BRT is generally well tolerated. Most radiation-related toxicities are grade ≤ 2 [86–88]. In an Italian multicenter study (*n* = 148), the dropout rate was only 3.2% with BRT, lower than chemotherapy bridging (10.2%) or no-bridging (21.2%). Moreover, a secondary analysis showed significantly fewer grade ≥ 3 CRS events in patients who received BRT compared to non-BRT groups [80].

#### Technical considerations in bridging radiotherapy

##### **Radiation field design**

Comprehensive fields covering all active lymphoma sites appear more effective than focal fields. Pinnix et al. and Manzar et al. observed improved 1-year PFS (50% vs. 28%) and OS (67% vs. 34%) with comprehensive radiotherapy [74, 89]. Mayo Clinic recommends a tailored approach: comprehensive fields for limited disease (< 5 nodal sites) and focal fields for diffuse disease (≥ 5 sites). PET-guided planning is standard, with GTV including PET-positive lesions, CTV adding a 0–1 cm margin, and PTV expanded by 0.5–1 cm per institutional protocols [90].

##### Radiation dose and fractionation

At present, considerable variability remains across studies and treatment centers regarding the selection of radiotherapy dose and fractionation, and no consensus has been established on the optimal BRT regimen. In Table 2, we provide a summary of the BRT regimens used in major studies. Overall, BRT has demonstrated a favorable safety profile and promising clinical utility in the context of CAR T-cell therapy. Its optimal application requires further clarification, and prospective studies are warranted.

**Table 2:** Summary of Bridging Radiotherapy Prior to CAR T-cell Therapy Across Studies

Author	Institution	Study period	Number of BRT patients	Bridging radiotherapy regimens	Response rates	Survival outcomes	CAR T–related toxicities
Sim et al.[87]	Moffitt Cancer Center	Dec 2017-Oct 2018	12	Median dose 20 Gy (range, 6–36.5 Gy); 2–4 Gy per fraction; Median fraction 5.5 (range, 3–14)	ORR 82%; CRR 45%	Median OS 6.3 months; 2-year OS 20%	CRS ≥ Grade 3: 27%
Imber et al.[86]	Memorial Sloan Kettering Cancer Center	NA	13	Median dose 20 Gy; 5 fractions	ORR 90%; CRR 70%	NA	CRS ≥ Grade 3: *n* = 1; ICANS ≥ Grade 3: *n* = 3
Wright et al.[88]	University of Pennsylvania	Aug 2018-Feb 2019	5	Median dose 37.5 Gy (range, 20–45 Gy); 2.2–4 Gy per fraction; Median fraction 15 (range, 5–20)	ORR 80%; CRR 60%	1-year PFS 20%; 1-year OS 80%	CRS ≥ Grade 3: 0%; ICANS ≥ Grade 3: 0%
Pinnix et al.[74]	MD Anderson Cancer Center	Nov 2017-Sep 2019	11	Median dose 35.2 Gy (range, 10–45 Gy)	ORR 100%; CRR 82%	Median PFS 8.9 months; Median OS not reached	CRS ≥ Grade 3: 0%; ICANS ≥ Grade 3: 27%
Kuhnl et al.[100]	10 UK centers	Dec 2018-Nov 2020	76	Median dose 20–40 Gy	ORR 63%; CRR 50%	1-year PFS 58%; 1-year OS 65%	CRS ≥ Grade 3: 11%; ICANS ≥ Grade 3: 13%
Yu et al.[80]	Tongji Medical College	Feb 2017-Oct 2020	29	Median dose 8 Gy (range, 2–20 Gy)	ORR 86%; CRR 52%	1-year PFS 47%; 1-year OS 61%	CRS ≥ Grade 3: 0%; ICANS ≥ Grade 3: 7%
Saifi et al.[101]	Mayo Clinic	2018–2021	35	Median dose 20 Gy	ORR 88%; CRR 59%	1-year EFS 48%; 1-year OS 72%	NA
Saifi et al.[102]	Mayo Clinic	2018–2020	14	Median dose 20 Gy (range, 15–36 Gy) ; Median fraction 5 (range, 3–24)	ORR 86%; CRR 50%	1-year PFS 47%; 1-year OS 67%	CRS ≥ Grade 2: *n* = 1; ICANS ≥ Grade 2: *n* = 3
Ladbury et al.[103]	City of Hope National Medical Center	Dec 2017-Mar 2021	12	Median dose 20 Gy; Median fraction size 2.25 Gy	CRR 67%	1-year PFS 75%; 1-year OS 92%	CRS ≥ Grade 3: 17%
Saif et al.[104]	Mayo Clinic	Apr 2018-May 2023	34	Median dose 26.5 Gy (range, 9.3–43.0 Gy)	ORR 91%; CRR 62%	2-year EFS 45%; 2-year OS 69%	NA
Shimizuguchi et al.[105]	Komagome Hospital	Jun 2020-Nov 2023	30	Median dose 40 Gy (range, 9.3–50 Gy)	NA	BT responders (CR/PR): 1-year PFS 78%, OS 94%	NA

Abbreviations: *BRT* bridging radiotherapy, *BT* bridging therapy, *CRR* complete response rate, *CRS* cytokine release syndrome, *EFS* event-free survival, *ICANS* immune effector cell–associated neurotoxicity syndrome, *NA* not applicable, *ORR* objective response rate, *OS* overall survival, *PFS* progression-free survival

## BT in pivotal clinical trials

BT strategies varied across pivotal clinical trials of different CAR T-cell products (Table 3). In the ZUMA-1 trial evaluating axi-cel, systemic chemotherapy as BT was not permitted [91], while the ZUMA-7 trial allowed corticosteroid-only BT, which was administered in 36% of patients [75]. In contrast, the JULIET trial of tisa-cel reported BT in 92% of patients, involving regimens that included rituximab (54%), gemcitabine (40%), etoposide (26%), dexamethasone (25%), cisplatin (19%), cytarabine (19%), ibrutinib (9%), and LEN (7%) [16]. In the BELINDA study, 83% of patients (135/162) received platinum-based BT; of these, 35.8% (*n* = 58) received a single cycle, and 47.5% (*n* = 77) received ≥ 2 cycles [92]. For lisocabtagene maraleucel, BT was administered in 59% of patients in the TRANSCEND trial and 63% in the TRANSFORM trial, with most patients receiving a single cycle of therapy [26, 93]. In the RELIANCE study evaluating relmacabtagene autoleucel (relma-cel), 44.2% (26/59) of patients received BT [94].

**Table 3:** BT in Pivotal Clinical Trials

CAR-T product	Pivotal clinical trial	BT patients (*n*, %)	BT strategies	Additional notes
Axi-cel[91]	ZUMA-1	-	-	Systemic chemotherapy not allowed as BT
Axi-cel[75]	ZUMA-7	65/180 (36%)	Allowed corticosteroid-only BT	-
Tisa-cel[16]	JULIET	92%	Rituximab 54%, gemcitabine 40%, etoposide 26%, dexamethasone 25%, cisplatin 19%, cytarabine 19%, ibrutinib 9%, LEN 7%	-
Tisa-cel[92]	BELINDA	135/162 (83%)	Investigator’s choice of four prespecified platinum-containing combination chemotherapy regimens	35.8% received 1 cycle, 47.5% received ≥ 2 cycles/regimens
Liso-cel[93]	TRANSCEND	159/269 (59%)	Systemic therapy, radiation therapy, or both were allowed	BT failed to reduce tumor burden in most patients
Liso-cel[26]	TRANSFORM	58/92 (63%)	BT allowed with one of the three defined salvage immunochemotherapy regimens–R-DHAP, R-ICE, R-GDP	91% received 1 cycle of BT; 9% received > 1 cycle; 9 patients achieved PET negativity before liso-cel; all showed sustained clinical benefit regardless of BT
Relma-cel[94]	RELIANCE	26/59 (44.2%)	Not reported	-

Abbreviations: *axi-cel* axicabtagene ciloleucel, *BT* bridging therapy, *liso-cel* lisocabtagene maraleucel, *relma-cel* relmacabtagene autoleucel, *R-DHAP* rituximab, dexamethasone, high-dose cytarabine, and cisplatin, *R-GDP* rituximab, gemcitabine, dexamethasone, cisplatin, *R-ICE* rituximab, ifosfamide, carboplatin, etoposide; tisa-cel, tisagenlecleucel

## BT in real-world studies

Real-world data indicate significantly higher utilization rates of BT compared to clinical trials (Table 4). In the U.S. Lymphoma CAR-T Consortium study (*n* = 298 DLBCL), 53% (*n* = 158) of patients received BT, including chemotherapy (54%), targeted therapy (10%), corticosteroids (23%), and radiotherapy (12%). The BT group demonstrated inferior 1-year OS compared to the non-BT group (56% vs. 81%, *P* < 0.001) [15]. Similarly, in another study, 55% (81/148) of patients underwent BT (systemic therapy/radiotherapy/combination), with 1-year OS significantly lower in the BT group than in the non-BT group (48% vs. 65%, *P* = 0.05). Notably, patients receiving BT were more likely to present with high-risk features, such as elevated IPI scores, bulky disease, and increased LDH levels [74]. In a Spanish cohort (*n* = 261), the BT rate reached 80%, predominantly with chemotherapy (60%) [95]. Another Spanish multicenter study of 412 patients showed BT rates of 77% in patients aged < 70 years and 85% in those ≥ 70 years (*P* = 0.371). Among the younger cohort, chemotherapy accounted for 88%, radiotherapy for 8%, and corticosteroids for 4%; in the older cohort, chemotherapy comprised 92% and radiotherapy 8% [96]. In Germany, a multicenter study involving 21 institutions reported a BT rate of 78%, primarily using platinum-based regimens [51]. Among 371 patients from Europe and the United States who received tisa-cel or axi-cel, 80% underwent BT (chemotherapy 57%, immunotherapy 7%, targeted therapy 8%, radiotherapy 17%). Bridging regimens that included immunotherapy were associated with the highest response rates (38%). BT was more frequently administered in patients receiving tisa-cel than axi-cel (94% vs. 73%, *P* < 0.0001), and the BT rate was significantly higher in the Europe compared to the United States (90% vs. 70%, *P* < 0.001) [97].

**Table 4:** BT Strategies in Real-World Studies

CAR-T product	Country/Region	BT patients (*n*, %)	BT regimens	Efficacy and survival outcomes
Axi-cel[15]	U.S. Lymphoma CAR-T Consortium	158/298 (53%)	Chemotherapy 54%, targeted therapy 10%, corticosteroids 23%, radiotherapy 12%	BT group showed lower 12-month OS than non-BT group (56% vs. 81%, *P* < 0.001)
Axi-cel[74]	United States	81/148 (55%)	Systemic therapy (*n* = 45), radiotherapy(*n* = 11), or combined modality therapy (*n* = 6))(19 patients did not proceed to axi-cel༉	1-year OS was lower in the BT group vs. non-BT (48% vs. 65%, *P* = 0.05); BT patients often had high-risk features
Axi-cel / Tisa-cel[95]	Spain	210/261(80%)	Chemotherapy-based in most cases (*n* = 127, 60%)	14% (*n* = 30) responded to BT (21 PR, 9 CR), mainly after chemotherapy; PD as best BT response correlated with poorer PFS
Axi-cel / Tisa-cel[96]	Spain	BT in 77% (< 70 yrs, *n* = 341) and 85% (≥ 70 yrs, *n* = 71) of 412 patients	In the younger group: chemotherapy 88%, radiotherapy 8%, corticosteroids 4%; in the older group: chemotherapy 92%, radiotherapy 8%.	Not reported
Axi-cel / Tisa-cel[51]	Germany	278/356 (78%)	Platinum-based chemoimmunotherapy (*n* = 67) or similar-intensity therapy (*n* = 71), Pola-based regimens (*n* = 71), other rituximab based chemoimmunotherapy (*n* = 33), radiotherapy (*n* = 30), immunotherapy (*n* = 12), steroids (*n* = 6)	BT group had higher IPI and elevated LDH; 22% achieved CR/PR (Pola-based: 34%); nonresponse to BT was associated with worse PFS and OS
Axi-cel / Tisa-cel[97]	Europe and United States	296/371 (80%)	Chemotherapy 57%, immunotherapy 7%, targeted therapy 8%, radiotherapy 17%	Immunotherapy-based BT showed the highest response rate (38%); BT use was more common with tisa-cel than axi-cel (94% vs. 73%) and higher in the Europe than the United States (90% vs. 70%).
Axi-cel / Tisa-cel[98]	United Kingdom	260/300 (87%)	Corticosteroids (*n* = 29), systemic therapies (*n* = 167), radiotherapy (*n* = 54), combined modality treatment (*n* = 10)	BT use and type were similar between axi-cel and tisa-cel cohorts
Axi-cel / Tisa-cel[11]	United Kingdom	326/375 (87%)	Chemotherapy 57%, radiotherapy 17%	CR/PR to BT reduced risk of progression or death by 42% post–CAR-T
Axi-cel / Tisa-cel[27]	France	59/61(97%)	Not reported	Not reported
Axi-cel / Tisa-cel[99]	Italy	393/485(81%)	Chemotherapy 41.5%, radiotherapy alone 21%, Pola-based regimens 24.2%	1-year OS/PFS by BT response: no BT (77%/47%), failure (54%/29%), PR (68%/40%), CR (82%/72%)

Abbreviations: *axi-cel* axicabtagene ciloleucel, *BT* bridging therapy, *CR* complete response, *CRR* complete response rate, *IPI* International Prognostic Index, *LDH* lactate dehydrogenase, *liso-cel* lisocabtagene maraleucel, *ORR* overall response rate, *OS* overall survival, *PD* progressive disease, *PFS* progression-free survival, *Pola* polatuzumab vedotin, *PR* partial response; tisa-cel, tisagenlecleucel

In a prospective UK study, the overall BT rate was 86.7%, with similar BT strategies observed between the tisa-cel and axi-cel groups [98]. Another UK study (*n* = 375) reported a BT rate of 87%, predominantly involving single-cycle chemotherapy. Patients who achieved CR/PR to BT had significantly better 1-year OS than non-responders (63.2% vs. 45.9%, *P* = 0.001), and multivariate analysis confirmed that BT response was independently associated with a lower risk of disease progression [11]. A French single-center study reported a BT rate as high as 97% [27]. In a prospective Italian study (*n* = 485), 81% (*n* = 393) of patients received BT, including chemotherapy (41.5%), radiotherapy alone (21%), and Pola-based regimens (24.2%). Stratification by BT response demonstrated significant prognostic value: 1-year OS rates were 77% (no BT), 54% (BT failure), 68% (BT with PR), and 82% (BT with CR) (*P* < 0.0001), while corresponding 1-year PFS rates were 47%, 29%, 40%, and 72%, respectively (*P* < 0.0001) [99].

## Conclusion

This review systematically summarizes current strategies and clinical applications of BT in the context of CAR T-cell therapy, incorporating evidence from both clinical trials and real-world studies. The selection of an appropriate bridging strategy—including chemotherapy, targeted agents, immunotherapeutic approaches, or radiotherapy—should be individualized based on disease subtype, tumor biology, and patient-specific factors such as treatment tolerance. In addition, different bridging strategies can modulate TME features, while the pre-treatment TME itself profoundly influences the efficacy and safety of CAR T-cell therapy. Therefore, a deeper understanding of how specific BT approaches reshape the lymphoma microenvironment—which in turn may alter CAR T-cell performance—remains an important area for future investigation. BT may be particularly beneficial for patients with high tumor burden, rapidly progressive disease, or critical symptoms, as it can help stabilize disease prior to CAR T-cell infusion. Candidates with chemo-sensitive disease, preserved organ function, and no active infections are generally more suitable for BT. Conversely, in patients with indolent or stable disease and low tumor burden, bridging may be unnecessary and could pose a risk of avoidable toxicity.

Overall, research on BT prior to CAR T-cell infusion has been steadily expanding, with an increasingly diverse array of therapeutic options now available. The preliminary evidence has supported the feasibility, efficacy, and safety of these approaches. However, several key questions remain to be addressed. These include the optimal use of BsAbs as bridging regimens, the timing and duration of BTKis, the safety and efficacy of reusing Pola as a bridging regimen in patients previously exposed to this agent, and the standardization of radiation field design, dosing, and fractionation strategies. Addressing these unresolved issues will require further robust clinical data.

With continued advancements in research and the ongoing refinement of bridging strategies, we can expect to more accurately identify patients most likely to benefit from BT, tailor individualized regimens accordingly, and facilitate a smoother transition to CAR T-cell infusion—ultimately aiming to improve patient survival outcomes.

## Data Availability

No datasets were generated or analysed during the current study.
